# Effect of method of administration on the oral health–related quality of life assessment using the Early Childhood Oral Health Impact Scale (ECOHIS-G)

**DOI:** 10.1007/s00784-021-03818-7

**Published:** 2021-02-11

**Authors:** Katrin Bekes, Cia Solanke, Tessa Waldhart, Julia Priller, Tanja Stamm

**Affiliations:** 1 Department of Paediatric Dentistry, University Clinic of Dentistry, Medical University of Vienna, Sensengasse 2a, 1090 Vienna, Austria; 2 Section for Outcomes Research, Center for Medical Statistics, Informatics, and Intelligent Systems, Medical University of Vienna, Spitalgasse 23, 1090 Vienna, Austria

**Keywords:** Oral health–related quality of life (OHRQoL), Early Childhood Oral Health Impact Scale (ECOHIS), Administration forms

## Abstract

**Objectives:**

The influence of the administration method used to collect oral health–related quality of life (OHRQoL) in children remains largely unknown. The aim of this study was to determine whether the OHRQoL information obtained using the Early Childhood Oral Health Impact Scale (ECOHIS) differed with different methods of data collection (face-to-face interview, telephone, or self-administered questionnaire).

**Materials and methods:**

The OHRQoL of 38 preschool children, aged 1 to 5 years, was measured using the German version of the ECOHIS. The instrument was administered to the caregivers of these children using three different methods, with an interval of 1 week between each administration. Test-retest reliability for the repeated ECOHIS-G assessments across the three methods of administration, agreement, and convergent validity was determined.

**Results:**

Kappa coefficients for agreement between two different methods of administration, respectively, ranged from moderate to substantial (0.47 to 0.65). Test-retest reliability was moderate (ICC 0.65–0.79).

**Conclusion:**

In conclusion, the three methods of administration (face-to-face interview, telephone interview, or self-administered questionnaire) of the ECOHIS-G were comparable in 1- to 5-year-old preschool children.

**Clinical relevance:**

All three methods of administration can be used to obtain valid and reliable OHRQoL information in German speaking countries.

## Introduction

Over the past several years, the concept of oral health–related quality of life (OHRQoL) has received great importance in the field of dentistry [1, 2]. Numerous patient-based measures have been developed to assess the effect of oral health problems on an individual’s physical, mental, and social health and well-being [3]. Initial instruments largely focused on adult populations. More recently, this interest has been extended to assessing OHRQoL in children and adolescents. The Early Childhood Oral Health Impact Scale (ECOHIS) is a questionnaire designed for adult caregivers that assesses the impact of oral health problems and related treatment experiences on the quality of life of preschool children and their families. The ECOHIS was originally developed by Pahel et al. [4] in the USA and has since been cross culturally adapted and successfully administered in studies performed across the globe [5–7].

Choosing the most suitable method of administration of an instrument is a crucial step in assessing one’s OHRQoL [8]. Two basic approaches exist on how the information is gathered—the (personal) interview and the (self-administered) questionnaire [9]. The personal interview is conducted either as a face-to-face interview or as a telephone interview, and the questionnaire is completed either manually on paper or electronically. Each of these methods of administration has their advantages and disadvantages in terms of applicability, patient burden, response rate, and cost [9]. Evidence suggests that respondents prefer filling out questionnaires over being interviewed [10]. However, this form of administration has been associated with lower response rates [11] and can only be administered to those with sufficient reading and comprehension skills.

In 2002, the Scientific Advisory Committee of the Medical Outcome Trust made the comparability of alternative forms of methods of administration one of the eight attributes for the assessment and review of health-related quality of life instruments [12]. Hence, studies investigating the effects of different administration methods of OHRQoL instruments are desired [13]. To date, there is no evidence proving whether the method of administration of the German version of the ECOHIS (ECOHIS-G) influences the OHRQoL assessment.

Therefore, it was the aim of this study to compare how the OHRQoL information obtained in preschool children aged 1 to 5 years using ECOHIS-G differed across the three administration methods: face-to-face interview, telephone interview, and self-administered questionnaire.

## Materials and methods

### Participants

Participants in the study were a convenience sample of 38 children aged 1 to 5 years (mean age 3.7 ± 1.0 years; 47.4% male) recruited from the Department of Pediatric Dentistry, University Clinic of Dentistry at the Medical University of Vienna, as well as from the “Martha Wolf” Kindergarten, Medical University of Vienna, Austria. At the time of enrollment in the study, the parents/guardians of the participants gave their signed informed consent. The study protocol was approved by the Institutional Review Board of the Medical University of Vienna (Reg. Nr.: 1822-2015).

### Data collection

OHRQoL was measured using the validated German version of the Early Childhood Oral Health Impact Scale (ECOHIS-G). The questionnaire consists of a total of 13 questions and is divided into two main parts, namely, the child impact section (9 items) and the family impact section (4 items). The child impact section (CIS) contains four subscales: symptom (one item), child-related function (four items), psychology (two items), and self-image/social interaction (two items). The family impact section (FIS) comprises two subscales: parental distress (two items) and family-related function (two items) [6].

The questions of the CIS and FIS inquire about the frequency of events in the child’s entire life and the responses are scored on an ordinal scale as follows: 0 = never, 1 = hardly ever, 2 = occasionally, 3 = often, 4 = very often. In every item, there exists an additional answer option “do not know” which was treated in the analysis like a missing answer. For participants with up to two missing responses in the child section and one in the family section, a score was imputed as an average of the previously calculated total. Participants with more than two and one missing responses in the child and family section respectively were excluded from the study [6]. Summing the response codes for the questionnaire items generates domain scores and an overall ECOHIS-G score. The instrument’s summary score ranges from 0 to 52; the scores for the child and family sections have a possible range from 0 to 36 and from 0 to 16, respectively. The higher the summary score, the worse the OHRQoL. Whereas, a score of 0 is indicative of absence of any problems. Furthermore, the ECOHIS includes two questions asking the caregiver for a global rating of the child’s oral health and overall well-being. The responses to these global ratings are as follows: excellent, very good, good, moderate, poor.

### Procedure

**Fig. 1:**
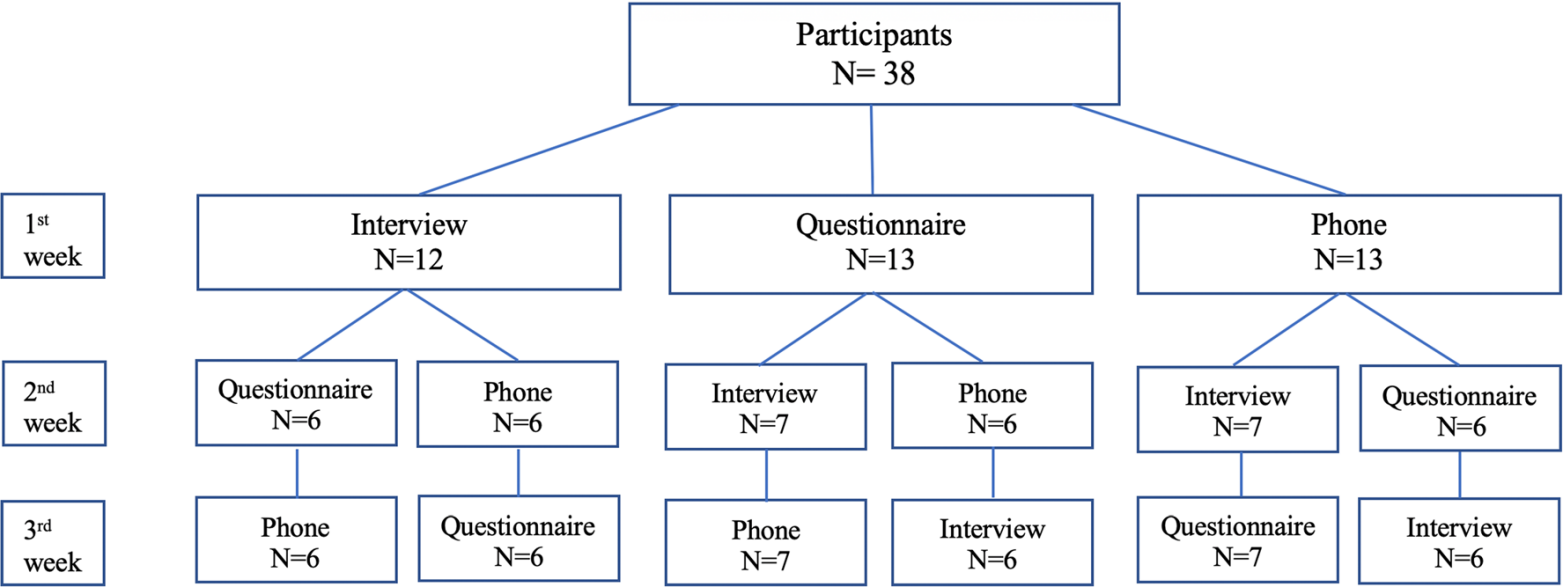
Flow chart displaying the number of participants in each group and study design

Three methods of ECOHIS-G administration were used: face-to-face interview conducted by a research assistant, self-administered questionnaire carried out manually, and telephone interview, also conducted by a research assistant. The personal interview took place at the Department of Paediatric Dentistry at the University Clinic of Dentistry as well as at the kindergarten. Both the face-to-face interview and the telephone interview were conducted by the one research assistant. The self-administered questionnaire was personally handed out to the guardians. The ECOHIS-G questionnaire was completed a total of three times by the participant’s parent/caregiver with a 1-week interval between each administration form. The order of the methods was block-randomized (i.e., the study subjects were randomized to blocks of six possible permutations of the three administration methods) (Fig. 1).

### Data analysis

The primary objective was to investigate the influence of different administration methods on the ECOHIS-G scores. In order to quantify the difference in the units of the OHRQoL, pairings of the three administration methods were compared using the method of Bland and Altman [14]. The term “bias” refers to the mean of these differences and the term “limits of agreement” refers to the reference interval (mean ± 1.96 × standard deviation). It is expected that the 95% limits include 95% of differences between the two measurement methods.

Additionally, test-retest reliability for the repeated OHRQoL assessment across the three administrations was assessed. This determines how much variation in ECOHIS-G scores is caused by true score reliability and therefore not by the influence of administration method and measurement error. It was assessed by calculating intraclass correlation coefficients (ICC) based on a one-way repeated-measures analysis of variance (ANOVA) [15]. An ICC value ≥0.80 reflected high reliability; ≥0.60, moderate reliability; and < 0.60, poor reliability [16]. In addition, we calculated correlation coefficients (Pearson or Spearman, depending on the distribution of the data) between two methods of administration of the ECOHIS-G sum score, respectively, and between each method of administration and the overall rating for oral and general health.

While reliability refers to consistency among methods of administration and is correlational in nature, agreement describes the interchangeability of methods of administration [17]. Cohen’s kappa coefficient measures agreement for categorical items by taking into account the possibility of the agreement occurring by chance [18]. We therefore calculated weighted kappa coefficients for two methods of administration, respectively. According to Cohen, kappa coefficients are interpreted as no agreement if values are ≤0, none to slight agreement for values ranging between 0.01 and 0.20, fair from 0.21 to 0.40, moderate from 0.41 to 0.60, substantial from 0.61 to 0.80, and perfect from 0.81 to 1.00. The data were analyzed using R (www.r-project.org). For sample size calculation, we estimated that 34 individuals were required for detecting an ICC of 0.6 with a desired confidence interval width of 0.2. We further estimated 10% drop outs and therefore aimed to include 38 participants for the test-retest analysis.

## Results

A total number of 38 preschool children, aged 1 to 5 years (mean age 3.7±1.0 years; 47.4% male), were recruited for this study. The parent’s ECOHIS-G responses produced a mean summary score of 3.3 ± 3.6 for the sample across all three modes of administration (Table 1). Males showed higher scores as compared to females 3.7 (± 3.7) vs. 2.8 (± 3.4). The majority of the parents perceived their children’s oral health as “excellent” and general health as “excellent” to “very good.” In regard to the oral and general health, moderate discrepancies were observed between the genders.

**Table 1:** ECOHIS-G mean summary scores (± SD) and distribution of self-rated oral and general health status for all participants stratified for gender

	All (*n* = 38)	Gender	
		Male (*n* = 18)	Female (*n* = 20)
ECOHIS-G	3.3 ± 3.6	3.7 ± 3.7	2.8 ± 3.4
Self-administered*	2.6 ± 2.9	2.7 ± 2.7	2.4 ± 3.0
Face-to-face interview	3.7 ± 4.2	4.6 ± 4.5	3.2 ± 3.9
Telephone interview	2.9 ± 3.2	3.5 ± 3.3	2.8 ± 3.2
Oral health			
Excellent	45.2%	47.0%	43.3%
Very good	43.6%	43.9%	43.3%
Good	11.2%	9.0%	13.4%
Moderate	-	-	-
Poor	-	-	-
General health			
Excellent	42.8%	43.9%	41.7%
Very good	42.9%	47.0%	38.3%
Good	13.5%	9.1%	18.3%
Moderate	0.8%	-	1.7%
Poor	-	-	-

*Self-administered questionnaire was completed on paper

For pairwise comparisons of methods of administration, the mean differences in summary scores ranged from − 1.19 to 0.38 points. The individual differences in summary scores obtained using different administration methods were found to be of considerable magnitude. These are indicated by the wide limits of agreement in Table 2. Agreement between the pairs of administration methods of the ECOHIS-G was moderate (ICC 0.65–0.79; Table 2).

**Table 2:** ECOHIS-G test-retest reliability and magnitude of differences in summary scores between different administration methods

Paired methods of administration	ICC (95% CI)	Mean difference (95% CI)	Limits of agreement
Self-administered versus interview	0.65 (0.40 to 0.79)	−1.13 (−2.32 to 0.05)	−8.19 to 5.93
Self-administered versus telephone	0.74 (0.54 to 0.85)	−0.34 (−1.26 to 0.57)	−5.79 to 5.11
Interview versus telephone	0.79 (0.64 to 0.88)	0.79 (−0.22 to 1.80)	−5.22 to 6.80

Spearman rank correlation coefficients between the ECOHIS-G sum scores of two methods of administration, respectively, showed significant correlations, as well as between the self-administered method and the general rating of oral health (Table 3). Weighted kappa coefficients for agreement between two different methods of administration, respectively, ranged from moderate to substantial (0.47 to 0.65) and are depicted in Table 4.

**Table 3:** Correlation coefficients (Spearman) between two methods of administration of the ECOHIS-G sum score, respectively, and between each method of administration and the overall rating for oral and general health. Significant coefficients (<0.05) are marked in bold

	Self-administered	Face-to-face interview	Telephone interview
Self-administered	-		
Face-to-face interview	**0.559**	-	
Telephone interview	**0.593**	**0.613**	-
Oral health	**−0.401**	**−**0.204	**−**0.110
General health	**−**0.249	**−**0.202	**−**0.312

**Table 4:** Weighted kappa coefficients for two methods of administration of the ECOHIS-G sum score, respectively, with 95% confidence boundaries

	Self-administered	Face-to-face interview	Telephone interview
Self-administered	-		
Face-to-face interview	0.47 (0.26 to 0.68)	-	
Telephone interview	0.57 (0.33 to 0.81)	0.65 (0.43 to 0.86)	-

## Discussion

Previous studies have proved that the method of administration of questions can potentially influence the answers [19]. However, the magnitude of this influence remains unknown for self-reported oral health status, especially in children. The aim of this study was to compare the results and the data quality of three methods of survey administration (face-to-face interview, self-administered questionnaire, and telephone interview) using the German version of the ECOHIS. Each study participant was tested using all three OHRQoL assessments, with a 1-week interval between each administration, which is in agreement with other studies also focusing on different methods of administration [9, 13].

We observed a slight difference in the ECOHIS mean summary scores across the three methods of administration. The mean score of the total ECOHIS for face-to-face interviews (3.7 ± 4.2) was higher than that for telephone interviews (2.9 ± 3.2) and the self-administered questionnaires (2.6 ± 2.9), indicating that the participants reported poorer OHRQoL in face-to-face interviews. As suggested by Sousa et al., this could have principally occurred as a result of the interaction with the interviewer [11].

Psychometric properties for ECOHIS-G information collected using different administration methods were similar to previously observed values in the German general population [6]. In that study’s reliability assessment, test-retest reliability was high (ICC 0.81). Interestingly, we reported slightly lower ICC values (0.65–0.79), indicating moderate reliability. Additionally, we measured agreement, which describes the interchangeability of the different methods of administration. Kappa coefficients for agreement between two different administration methods respectively ranged between 0.47 and 0.65 and were deemed moderate to substantial. Convergent validity was demonstrated by calculating the Spearman rank correlation coefficients. This type of validity evaluates the degree to which two or more measures that theoretically should be related to each other are, in fact, observed to be related to each other. Spearman rank correlation coefficients between the ECOHIS-G sum scores of two administration methods, respectively, showed significant correlations, as well as between the self-administered method and the general rating of oral health.

The higher ECOHIS mean values across all three methods of administration for males may be a random effect. There is no prior study evaluating the methods of survey administration of the ECOHIS which has assessed this parameter. However, Malter et al. observed higher CPQ-G 11-14 mean values for males using the self-administered questionnaire [13]. The authors did not find these results when they reported the population-based norms using the same administration method [20]. The authors also mentioned that, from a point of clinical relevance, a gender difference of 2 or 3 CPQ points is not meaningful.

To our knowledge, only one published study has investigated the effect of the administration method of the OHRQoL assessment with the ECOHIS. Ortiz et al. assessed the psychometric properties and the level of agreement between the face-to-face and telephone method of administration for children up to 5 years [21]. In this study, the Brazilian version of the ECOHIS (13 items) was used and this questionnaire was administered to the same patients/caregivers in the two different methods with a 2-week interval between them. The authors found acceptable psychometric properties for both the face-to-face and the telephone administered versions of the ECOHIS. Agreement between the two methods of administration was excellent (ICC 0.91–0.93). The two methods of administration agreed on nearly all the domains of the ECOHIS, except the child section function, total child section, and family section function, which resulted in differences in summary scores of the face-to-face and telephone administration methods. The authors speculated this difference to be a result of the questionnaire, and not the individual or the method applied. The authors concluded that both the administration methods of the ECOHIS demonstrated satisfactory psychometric properties and a high level of agreement. The results of our study pertain to these two administration methods as well as the self-administered questionnaire. In contrast to the results of Ortiz, we found slightly lower ICC values ranging between 0.65 and 0.79. Nevertheless, when agreement statistics were applied according to the guidelines [16], the observed ICC implied moderate agreement.

We also compared our results with previous OHRQoL findings in German children and adolescents assessed with the German version of the Child’s Perception Questionnaire (CPQ-G 11-14). In this study, the authors investigated the same three methods of administration (face-to-face interview, telephone interview, and self-administered questionnaire) in German children and adolescents aged 11–14 years. The psychometric core properties of the CPQ-G 11-14 scores obtained by different methods were similar and are in accordance with our study results.

As per the design of our study, the caregivers answered the ECOHIS-G questionnaire a total of three times (with an interval of 1 week), using each of the three methods. This study design offers the advantage of allowing a direct comparison to be made in the same participants, thus avoiding bias. Studies on test-retest reliability regarding health-related quality of life measures employ varying intervals between test administrations, ranging from 10 min to 1 month [21, 22].

So far, OHRQoL information in general, and ECOHIS data specifically, has been collected mainly using self-administered questionnaire. Our results help conclude that the psychometric properties of the ECOHIS were not affected by the method of administration. This allows researchers to choose the most appropriate method suitable to them, having considered the financial and feasibility aspect of the various methods available. In the present study, all the three methods investigated were reliable and valid in the assessment of OHRQoL using the German version of the ECOHIS. As suggested by Reissmann et al., although comparable results can be expected irrespective of the method of administration used, it is crucial that the administration method be held constant as much as possible for repeated OHRQoL assessment [9]. Otherwise, the results should be adjusted for the potential bias system [23].

Future qualitative, feasibility, or user experience studies should evaluate what kind of interview was most appropriate in the view of the parents. This might be important for clinical setting, because the most comfortable patient-reported outcome might provide best information and compliance.

## Conclusion

In conclusion, all three methods of administration of the ECOHIS-G demonstrated satisfactory psychometric core properties (reliability and validity) and a good level of agreement.
